# Partial palivizumab prophylaxis and increased risk of hospitalization due to respiratory syncytial virus in a Medicaid population: a retrospective cohort analysis

**DOI:** 10.1186/1471-2431-14-261

**Published:** 2014-10-13

**Authors:** Leonard R Krilov, Anthony S Masaquel, Leonard B Weiner, David M Smith, Sally W Wade, Parthiv J Mahadevia

**Affiliations:** Children’s Medical Center, Winthrop University Hospital, Mineola, NY USA; State University of New York Stony Brook School of Medicine, Stony Brook, New York, NY USA; MedImmune, Gaithersburg, MD USA; Upstate Golisano Children’s Hospital, State University of New York, Upstate Medical University, Syracuse, NY USA; Truven Health Analytics, Washington, DC USA; Wade Outcomes Research and Consulting, Salt Lake City, UT USA

**Keywords:** Prophylaxis, Respiratory syncytial virus, Palivizumab, Non-compliance

## Abstract

**Background:**

Infection with respiratory syncytial virus (RSV) is common among young children insured through Medicaid in the United States. Complete and timely dosing with palivizumab is associated with lower risk of RSV-related hospitalizations, but up to 60% of infants who receive palivizumab in Medicaid population do not receive full prophylaxis. The purpose of this study was to evaluate the association of partial palivizumab prophylaxis with the risk of RSV hospitalization among high-risk Medicaid-insured infants.

**Methods:**

Claims data from 12 states during 6 RSV seasons (October 1^st^ to April 30^th^ in the first year of life in 2003–2009) were analyzed. Inclusion criteria were birth hospital discharge before October 1^st^, continuous insurance eligibility from birth through April 30^th^, ≥ one palivizumab administration from August 1^st^ to end of season, and high-risk status (≤34 weeks gestational age or chronic lung disease of prematurity [CLDP] or hemodynamically significant congenital heart disease [CHD]). Fully prophylaxed infants received the first palivizumab dose by November 30^th^ with no gaps >35 days up to the first RSV-related hospitalization or end of follow-up. All other infants were categorized as partially prophylaxed.

**Results:**

Of the 8,443 high-risk infants evaluated, 67% (5,615) received partial prophylaxis. Partially prophylaxed infants were more likely to have RSV-related hospitalization than fully prophylaxed infants (11.7% versus 7.9%, p< 0.001). RSV-related hospitalization rates ranged from 8.5% to 24.8% in premature, CHD, and CLDP infants with partial prophylaxis. After adjusting for potential confounders, logistic regression showed that partially prophylaxed infants had a 21% greater odds of hospitalization compared with fully prophylaxed infants (odds ratio 1.21, 95% confidence interval 1.09-1.34).

**Conclusions:**

RSV-related hospitalization rates were significantly higher in high-risk Medicaid infants with partial palivizumab prophylaxis compared with fully prophylaxed infants. These findings suggest that reduced and/or delayed dosing is less effective.

**Electronic supplementary material:**

The online version of this article (doi:10.1186/1471-2431-14-261) contains supplementary material, which is available to authorized users.

## Background

Annually between 75,000 and 250,000 hospitalizations in the United States (U.S.) may be attributed to infection with respiratory syncytial virus (RSV) among young children [1]. High-risk populations for severe RSV disease include premature infants ≤35 weeks gestational age (wGA), children with chronic lung disease of prematurity (CLDP), and children with hemodynamically significant congenital heart disease (CHD) [2, 3]. RSV was responsible for 1.7 million office visits, 402,000 emergency room visits, 236,000 hospital outpatient visits, and between 75,000 and 125,000 hospital admissions in children under 5 years of age in the U.S. in 2000 [4]. The burden of RSV disease is well-documented in high-risk populations in Medicaid programs. In one study, the RSV hospitalization rates per 1000 children less than 1 year of age were 388 for infants with bronchopulmonary dysplasia (BPD), 92 for infants with CHD, and 57 to 70 for premature infants depending on wGA, compared to a rate of 30 for term infants without medical risk factors [5]. Others have found a higher risk of RSV hospitalization in Medicaid compared to non-Medicaid infants [6, 7]. Complete and timely dosing with palivizumab is associated with lower risk of RSV-related hospitalizations, yet research shows that up to 60% of infants who received palivizumab in Medicaid populations do not receive full prophylaxis [2, 3, 8–10].

Per the package insert, palivizumab dosing consists of monthly intramuscular injections administered throughout the RSV season [2, 11]. Mean half-life of palivizumab is approximately 20 days and compliance to the monthly dosing schedule is important to sustaining sufficient RSV-neutralizing antibody levels throughout the therapeutic period. Efficacy of less frequent dosing has not been established [2, 3, 12].

The objective of the current study was to evaluate the association between partial palivizumab prophylaxis and the risk of RSV hospitalizations in a large population of high-risk infants with Medicaid coverage.

## Methods

### Data source

Study data was obtained from the MarketScan Medicaid Multi-State Database*®* (2003–2009) which contained the pooled experience of 12 million Medicaid enrollees from 12 geographically dispersed U.S. states. This database includes records of plan eligibility, inpatient and outpatient services, outpatient prescription drugs, and long-term care. Data are fully compliant with the Health Insurance Portability and Accountability Act of 1996. Because this study did not involve the collection, use, or transmittal of individually identifiable data, Institutional Review Board review was not required.

### Study population selection and analysis periods

All infants born between May 1^st^ and September 30^th^ in 2003 through 2008 whose database records could be linked to their birth hospitalization record were selected. This selection window intentionally excludes infants born during RSV season because dosing of palivizumab during birth hospital stay cannot be identified in claims data. Potential study patients were required to have continuous medical and pharmacy benefits from the birth date (index date) through April 30^th^ of the first year of life, to have been discharged from the birth hospitalization prior to October 1^st^ of the birth year, and to have received at least one dose of palivizumab. The start of the RSV season varies across the US, and we included only infants whose first dose was in August or later. We focused on high-risk infants (preterm infants ≤34 wGA, infants with CLDP or with hemodynamically significant CHD regardless of wGA). While on-label palivizumab use includes 35 wGA infants, the ICD-9-CM code combines this group with 36 wGA thus precluding their identification for our study.

The time between birth and the first palivizumab administration was defined as the pre-period. While RSV season is traditionally defined as November through March, we allowed an additional month on either side since our study covers a wide geographic range and multiple seasons. The October start allows for early seasons and the April end allows for late seasons. RSV hospitalizations were examined during RSV season (Observation Period 1), defined as October 1^st^ to April 30^th^ of the first year of life. Observation Period 2 was of variable length and defined as the time after the first palivizumab administration through the end of RSV season.

### Demographic and clinical characteristics

Demographic characteristics measured at birth included gender, race (white, black, Hispanic, other/unknown), urban or rural residence, presence of capitated services, and Medicaid-reported basis of eligibility as blind/disabled.

Clinical characteristics measured at the birth hospitalization included presence of a neonatal intensive care unit (NICU) admission and length of hospitalization stay (LOS). Birth month, birth type (singleton, multiplets, unknown), wGA (<33, 33–34, other [i.e., >34 with CLDP/CHD], and unknown), and birth weight (<500 grams, 500–999 grams, 1000–1499 grams, 1500–1999 grams, 2000–2499 grams, 2500+ grams, low birth weight unspecified, and missing) were also obtained.

CLDP, hemodynamically significant CHD, and other comorbidities of interest (Additional file 1) occurring in the pre-period were reported. Comorbid conditions were identified by the presence of a non-diagnostic claim with a relevant ICD-9-CM diagnosis code. Our CLDP definition was consistent with the American Thoracic Society definition, and in addition to a relevant diagnosis, we required use of a CLDP-specific medication or oxygen before the first palivizumab claim [13]. Similarly, a relevant medication in conjunction with a CHD-specific procedure or relevant ICD-9-CM diagnosis code identified hemodynamically significant CHD infants. The inclusion of infants with hemodynamically significant CHD is consistent with labeled indications for palivizumab in the U.S.

Because healthcare utilization is a proxy for health status, infants with emergency department (ED) visits or inpatient admissions for any cause prior to the start of the RSV season or the first palivizumab dose were identified and these data were used as covariates in multivariate analyses.

### Palivizumab prophylaxis

Infants in the study population were classified as receiving partial or full prophylaxis based on palivizumab doses received up to the date of the first RSV-related hospitalization or the end of follow-up, whichever occurred first. Consistent with Frogel et al., infants who obtained the first palivizumab dose by November 30^th^, with no more than 35 days between consecutive doses were considered fully prophylaxed [6]. Palivizumab claims within 7 days of each other (21% of all claims) were considered billing artifacts (e.g., result of separate billing for drug versus administration) and treated as a single dose. Age at first dose, the total number of doses (mean, median, range), and the number and percentage of infants with first dose after November 30^th^ were determined. Using all available data, we also determined the number and percentage of infants with ≥1 gap (>35 days between consecutive doses), the timing of gaps in the dosing sequence, and the number of days between doses for infants with ≥1 gap (mean, median, range). We also examined the percentage of infants with<5 doses and ≥5 doses, and computed the percentage of infants in each of these two groups who had therapy gaps.

### Hospitalization for RSV-related conditions

Hospitalizations for RSV-related conditions were examined during the pre-period, Observation Period 1, and Observation Period 2. RSV-related hospitalizations were defined by ICD-9-CM codes for RSV (079.6); acute bronchiolitis due to RSV (466.11); pneumonia due to RSV (480.1); acute bronchitis (466.0); acute bronchiolitis due to other infectious organisms (466.19); viral pneumonia, unspecified (480.9); bronchopneumonia, organism unspecified (485.xx); and pneumonia, organism unspecified (486.xx). Inpatient claims for unspecified bronchiolitis, with evidence of influenza or other bacterial pneumonia (ICD-9-CM codes: 481, 482.xx or 487.xx) within ±3 days of the hospitalization were excluded.

#### Pre-period RSV-related hospitalizations

Since infants hospitalized for RSV prior to receiving their first palivizumab administration may be clinically different from and have higher costs than other infants, in the multivariate analyses, we controlled for pre-period RSV-related hospitalizations. In sensitivity analyses, we also examined multivariate results after excluding infants who had any RSV-related hospitalization that occurred prior to the first palivizumab dose and before December 1.

#### RSV-related hospitalization in observational periods

For Observational Period 1, we determined the incidence of RSV-related hospitalization, the mean number of hospitalizations among infants with at least one such hospitalization, and age at first admission. We also examined the severity of RSV-related hospitalization using mean LOS, and admission to intensive care unit (ICU) or use of mechanical ventilation or supplemental oxygen. For Observational Period 2, we calculated the rate of RSV-related hospitalizations per 100 infant seasons. The number of infants with an RSV-related hospitalization following their first palivizumab dose (numerator) was divided by the total number of person-days in the observed seasons divided by 210 days (October 1-April 30) or the length of an RSV season. This result was multiplied by 100 to set 100 infant seasons. Person-days was the total number of follow-up days after first dose for the group overall (censored at 210 days or end of season).

### Analyses

Categorical variables were presented as the number and percentage; continuous variables were summarized by the mean and standard deviation (SD). Chi-square tests were used to evaluate the statistical significance of difference for categorical variables; t-tests and ANOVA were used for normally distributed continuous variables. Nonparametric Wilcoxon and Kruskal-Wallis tests were used for continuous variables that were not normally distributed.

Correlates of full prophylaxis were assessed using logistic regression with logit link and binomial variance function. Stepwise regression (inclusion and exclusion threshold p< 0.05) was used to select variables for the final model, results of which were used to construct propensity score-based weights for the study population. These weights were then used to balance differences in the characteristics of fully and partially prophylaxed infants in the weighted models.

Unweighted and weighted estimates for the risk of in-season RSV-related hospitalization were generated using logistic regression with logit link and binomial variance function. Covariates included demographics, comorbidities, and other potentially confounding variables, in addition to prophylaxis status. For sensitivity analysis, these models were also run to assess the risk of hospitalizations with an explicit RSV diagnosis code. All analyses were completed using SAS® software, version 9.2 (SAS Institute, Inc., Cary NC, USA).

## Results

**Figure 1:**
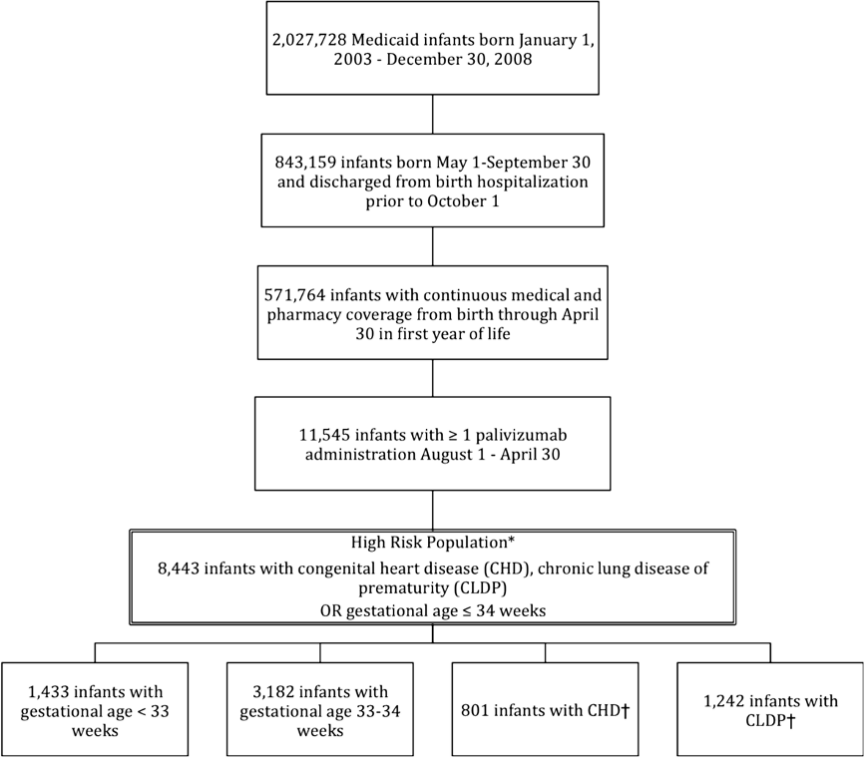
**Patient selection.** *2,648 infants coded as premature, unknown gestational age; 735 infants coded as live birth gestational age unknown. †Groups are not mutually exclusive. CHD and CLDP infants are also included in premature groups<33 and 33–34 weeks gestational age. CHD: congenital heart disease; CLDP: chronic lung disease of prematurity.

A total of 11,545 infants met the study criteria (Figure 1). Of these infants, 8,443 were identified as high-risk based on gestational age ≤34 weeks or presence of CLDP or CHD regardless of wGA.

### Demographic and clinical characteristics of infants based on palivizumab compliance

Two-thirds (5,615/8,443) of the sample were partially prophylaxed (Table 1). Compared with fully prophylaxed infants, these infants were more likely to be black or Hispanic (p< 0.001), reside in urban areas (p< 0.001), belong to capitated health plans (p< 0.001), and less likely to have blind/disabled eligibility for Medicaid (p= 0.043), be a multiplet (p< 0.001), or have NICU admission at birth (p= 0.002). Partially prophylaxed infants were also more likely to have CLDP (p< 0.001) and CHD (p< 0.001) and to experience ED visits or inpatient admissions for RSV or other causes prior to the first palivizumab dose (p< 0.001). Proportions of infants with additional specific comorbid conditions are presented in Additional file 2.

**Table 1:** **Characteristics of study population stratified by palivizumab prophylaxis through end of first RSV season**

Characteristic	Fully prophylaxed infants (N= 2828)		Partially prophylaxed infants (N= 5615)		p-value (fully versus partially prophylaxed)
	Number	Percent	Number	Percent	
**Demographics**					
**Sex**					
Male	1468	51.9%	2989	53.2%	0.250
Female	1360	48.1%	2626	46.8%	
**Race**					
White	1283	45.4%	2029	36.1%	<0.001
Black	795	28.1%	1759	31.3%	
Hispanic	235	8.3%	724	12.9%	
Other/unknown	515	18.2%	1103	19.6%	
**Population density**					
Urban	1898	67.1%	4230	75.3%	<0.001
Rural	924	32.7%	1370	24.4%	
Unknown	6	0.2%	15	0.3%	
**With capitated insurance**	545	19.3%	1724	30.7%	<0.001
**With basis of eligibility blind/disabled**	301	10.6%	520	9.3%	0.043
**Comorbidities of Interest**					
**Any CLDP**	192	6.8%	609	10.8%	<0.001
**Any CHD**	378	13.4%	864	15.3%	0.013
**Other comorbidity**	1565	55.6%	3171	56.3%	0.322
**Birth-related metrics**					
**Birth month**					
May	629	22.2%	1267	22.6%	<0.001
June	737	26.1%	1256	22.4%	
July	724	25.6%	1311	23.3%	
August	523	18.5%	1210	21.5%	
September	215	7.6%	571	10.2%	
**Birth type**					
Singleton	2092	74.0%	4199	74.8%	<0.001
Multiplets	575	20.3%	982	17.5%	
Unknown	161	5.7%	434	7.7%	
**Gestational age (weeks)**					
<33	455	16.1%	978	17.4%	0.125
33-34	1171	41.4%	2011	35.8%	<0.001
Other*	73	2.6%	156	2.7%	0.599
Live birth/premature, gestational age unknown	1074	38.0%	2309	41.1%	0.005
**Birth weight (grams)**					
<500	14	0.5%	25	0.4%	<0.001
500-999	368	13.0%	820	14.6%	
1000-1499	836	29.6%	1496	26.6%	
1500-1999	760	26.9%	1329	23.7%	
2000-2499	257	9.1%	487	8.7%	
2500+	75	2.7%	154	2.7%	
Low birth weight, not otherwise specified	205	7.2%	471	8.4%	
Missing	313	11.1%	833	14.8%	
**Birth hospitalization**					
**NICU admission**	2627	92.9%	5103	90.9%	0.002
**Mean/SD length of stay (days)**	28.9	24.9	27.0	24.7	0.001
**Utilization prior to first palivizumab dose**					
**Any ED visit or inpatient admission** ^**†**^	1031	36.5%	2590	46.1%	<0.001
**Any RSV-related hospitalization** ^**†**^	83	2.9%	334	5.9%	<0.001
**Palivizumab dosing**					
**Age at first dose (days)**	109.4	40.7	133.5	56.2	<0.001
**Number of doses**					
Mean/SD	6.3	1.2	3.8	1.8	<0.001
Median	6		4		
Range	3-13		1-11		

*Greater than 34 weeks gestational age with CLDP/CHD.

^†^Prior to first palivizumab dose and excluding birth hospitalization.

*CHD*: Congenital heart disease; *CLDP*: Chronic lung disease of prematurity; *NICU*: Neonatal intensive care unit; *ED*: Emergency Department; *SD*: Standard deviation.

### Palivizumab dosing patterns

Between birth and the end of their first RSV season, fully prophylaxed infants averaged 6.3 doses compared with 3.8 for partially prophylaxed infants (p< 0.001). Of the 5,615 partially prophylaxed infants, 3,408 (60.7%) had ≥1 gap in palivizumab dosing and 1,877 (33.4%) received the first palivizumab dose after November 30^th^. The majority of dosing gaps occurred before the 3^rd^ dose, (36.8% of gaps occurred between the first and second doses; 25.5% between the second and third doses; 20.2% between third and fourth doses; 11.8% between fourth and fifth doses; 5.8% between fifth and sixth doses). Among partially prophylaxed infants with at least one dosing gap, an average of 56.5 days elapsed between first and second doses; 51.7 days between second and third doses; 48.0 days among third and fourth doses; 46.4 days between fourth and fifth doses; and 44.0 days between fifth and sixth doses.

**Figure 2:**
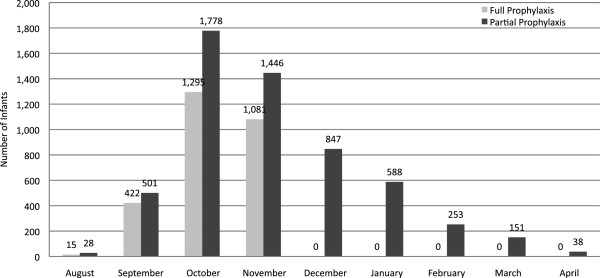
**Month of first palivizumab administration (N= 8,443).**

The proportion of infants with partial prophylaxis was higher among African Americans (68.9%; p< 0.001) and Hispanics (75.5%; p< 0.001) compared with Caucasians (61.3%). African American and Hispanic partially prophylaxed infants received significantly (p< 0.001) fewer doses compared with Caucasians (Table 2). Dosing gaps were also longer for African Americans and Hispanics compared with Caucasians, though the difference was not significant for African Americans (p= 0.063). Finally, partial prophylaxis was more common in capitated plans for African Americans and Caucasians compared with non-capitated plans. Figure 2 presents the distribution by month of the first palivizumab administration. A substantial number of partially prophylaxed infants did not receive palivizumab until long after the start of RSV season.

**Table 2:** **Partial prophylaxis rates among ethnic/racial minorities**

Dosing characteristics	Caucasian reference group (N= 3312)	African American (N= 2554)	Hispanic (N= 959)
Total population			
Partial prophylaxis, %	61.3	68.9 (p< 0.001)	75.5 (p< 0.001)
Mean (SD) doses among partially prophylaxed	3.9 (1.8)	3.8 (1.8) (p= 0.008)	3.5 (1.7) (p< 0.001)
Mean days between doses among gap >35 days	50.3 (19.5)	51.8 (19.2) (p= 0.063)	54.6 (20.2) (p< 0.001)
First palivizumab dose after November 30, %	31.8	28.0 (p= 0.869)	50.8 (p< 0.001)
Infants with capitated coverage			
Number of infants	787	863	190
Partial prophylaxis in capitated plans, %	71.4	80.2 (p< 0.001)	74.7 (p= 0.359)
Mean (SD) doses among noncompliant	3.8 (1.7)	3.6 (1.6) (p= 0.194)	3.2 (1.8) (p< 0.0001)
Mean days between doses among gap >35 days	50.5 (18.6)	52.9 (20.9) (p= 0.094)	55.0 (16.7) (p= 0.074)
First palivizumab dose after November 30, %	35.4	34.0 (p= 0.370)	57.0 (p< 0.001)
Infants with non-capitated coverage			
Number of infants	2525	1691	769
Partial prophylaxis in capitated plans, %	58.1	63.1 (p= 0.001)	75.7 (p< 0.001)
Mean (SD) doses among noncompliant	4.0 (1.9)	3.8 (1.9) (p= 0.073)	3.6 (1.7) (p< 0.001)
Mean days between doses among gap >35 days	50.1 (19.8)	51.0 (18.0) (p= 0.353)	54.6 (20.9) (p= 0.001)
First palivizumab dose after November 30, %	30.4	24.2 (p= 0.040)	49.3 (p< 0.001)

### RSV-related hospitalization rates

In our sample, there were a total of 1,368 RSV-related hospitalizations. More than one-third (36.8%) of RSV-related hospitalizations occurred prior to the first palivizumab dose. The percentage of RSV-related hospitalizations that occurred between doses was highest early in the dosing sequence (21.4% between the first and second doses; 8.6% between the second and third doses; 7.5% between the third and fourth doses; 5.2% between fourth and fifth doses and 4.0% between the fifth and sixth doses). Palivizumab dosing was continued for 83.6% of infants after RSV-related hospitalization.

**Figure 3:**
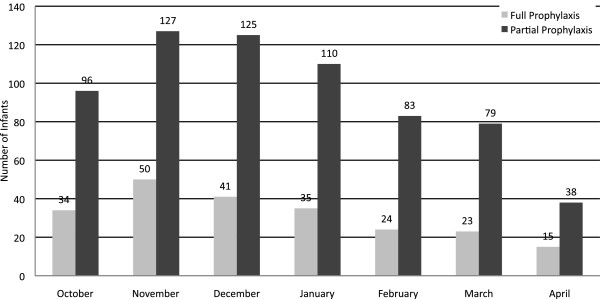
**Month of first RSV-related hospitalization by compliance status (N= 8,443).** RSV: respiratory syncytial virus.

**Figure 4:**
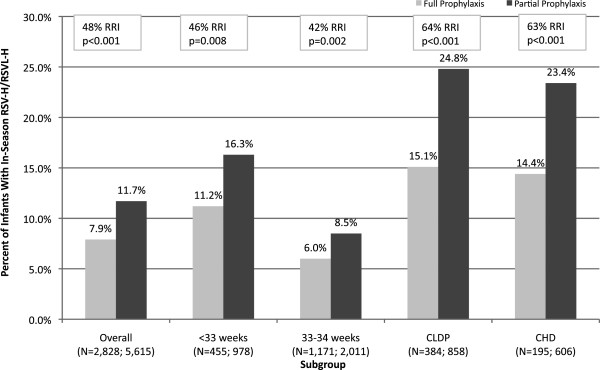
**Increase risk of RSV-related hospitalization among noncompliant infants in a medicaid population.** RSV: respiratory syncytial virus; RRI: relative risk reduction; CHD: congenital heart disease; CLDP: chronic lung disease of prematurity.

In unadjusted analyses (Table 3) during Observational Period 1, a significantly higher percentage of partially prophylaxed infants (11.7%) were hospitalized with an RSV-related illness during the season compared to fully prophylaxed infants (7.9%) (p< 0.001). In Observational Period 2, the RSV-related hospitalization rate per 100 infant seasons was 14.5 for partially prophylaxed infants compared with 10.0 for fully prophylaxed infants (p<0.001). The frequency of RSV-related hospitalizations was higher for partially prophylaxed infants throughout the RSV season (Figure 3). Figure 4 presents the unadjusted relative risk increase (RRI) for RSV-related hospitalizations among partially prophylaxed infants. The RRI was 48% (p< 0.001) for the partial prophylaxis cohort overall, and varied from 42% (p= 0.012) to 64% (p< 0.001) depending on gestational age or type of comorbidity. Among infants with RSV-related hospitalizations, partially prophylaxed infants had longer hospital stays and were more likely to be admitted to the ICU or to receive mechanical ventilation or supplemental oxygen compared with fully prophylaxed infants (p< 0.001 for both) (Table 3).

**Table 3:** Hospitalizations for RSV-related conditions among fully prophylaxed and partially prophylaxed infants*

Measure	Fully prophylaxed infants (N= 2828)		Partially prophylaxed infants (N= 5615)		p-value (fully versus partially prophylaxed)
	Number/mean	Percent/SD	Number/mean	Percent/SD	
Number of infants with ≥1 in-season hospitalization^†^	222	7.9%	658	11.7%	<0.001
Mean hospitalizations among patients with at least 1 hospitalization	1.3	0.6	1.3	0.7	1.000
Age (days) at first admission	180.4	71.3	174.7	66.4	0.278
Mean length of stay (days)	4.5	3.2	6.4	7.4	<0.001
Number of infants admitted to ICU or receiving mechanical ventilation or supplemental oxygen	12	6.3%	83	16.8%	<0.001
RSV hospitalizations per 100 seasons^‡^	10.0	40.3	14.5	54.7	<0.001

*Includes all infants in study population (N= 8443); first palivizumab dose may have occurred prior to or during RSV season.

^†^RSV season= October 1 through April 30.

^‡^Season rate calculation= (Number of infants with RSV hospitalization in 210 days after first dose or between first dose through April 30, whichever comes first) divided by (days evaluated divided by 210) times 100. We assumed an RSV season of October-April which corresponds to 210 days.

### Multivariate analyses

In weighted logistic regression, partially prophylaxed infants had significantly higher odds of in-season RSV-related hospitalization compared to fully prophylaxed infants [odds ratio (OR) 1.21; 95% confidence interval (CI) 1.09-1.34] (Table 4). Results were very similar [OR 1.28; 95% CI 1.09-1.51] when the outcome was restricted to hospitalizations with an explicit RSV diagnosis code. Compared with Caucasian race, “other” race was associated with an increased risk of hospitalization. Gender (male), residence (rural), type of health coverage (capitated) and older age (>3 months versus ≤3 months) at start of RSV season were each associated with an increased risk. Odds of RSV-related hospitalization during the RSV season were also higher for infants with CLDP, CHD, other comorbidities, NICU days during birth hospitalization, RSV-related hospitalizations prior to RSV season and emergency or inpatient care prior to first dose.

**Table 4:** **Propensity score weighted logistic regression of RSV-related hospitalization**

Characteristic	Effect comparison	Full study population (N= 8443)			Subgroup population* (N= 8106)		
		Odds ratio	95% confidence limits		Odds ratio	95% confidence limits	
Full prophylaxis	No vs. Yes	**1.207**	**1.088**	**1.339**	**1.253**	**1.115**	**1.408**
Gender	Male vs. Female	**1.163**	**1.047**	**1.291**	**1.19**	**1.058**	**1.338**
Race	Black vs. White	0.956	0.835	1.095	**0.742**	**0.634**	**0.869**
	Hispanic vs. White	1.132	0.947	1.354	1.151	0.947	1.4
	Other vs. White	**1.214**	**1.044**	**1.412**	**1.219**	**1.034**	**1.438**
Population density	Rural vs. Urban	**1.375**	**1.222**	**1.549**	**1.378**	**1.208**	**1.573**
	Unknown vs. Urban	1.448	0.559	3.751	1.939	0.759	4.954
Blind or disabled basis of eligibility	Characteristic Present vs. Absent	1.012	0.846	1.212	0.953	0.781	1.163
Capitation indicator	Characteristic Present vs. Absent	**1.269**	**1.121**	**1.437**	**1.292**	**1.124**	**1.486**
Birth year	Year 2004 vs. 2003	0.914	0.784	1.064	0.889	0.75	1.053
	Year 2005 vs. 2003	0.847	0.714	1.006	0.826	0.683	1
	Year 2006 vs. 2003	0.899	0.742	1.09	0.894	0.724	1.105
	Year 2007 vs. 2003	**0.751**	**0.616**	**0.914**	**0.646**	**0.515**	**0.812**
	Year 2008 vs. 2003	0.929	0.771	1.119	0.865	0.702	1.067
Age at onset of RSV season	>3 months vs. ≤3 months	**1.284**	**1.153**	**1.43**	1.127	0.999	1.271
Gestational age	<32 weeks vs. 33–34 Weeks	1.012	0.734	1.395	0.977	0.667	1.433
	35-36 weeks† vs. 33–34 Weeks	**2.523**	**1.469**	**4.336**	**2.53**	**1.384**	**4.626**
	37+ weeks† vs. 33–34 Weeks	**1.394**	**1.191**	**1.633**	**1.548**	**1.303**	**1.839**
	Gestational Age Unknown vs. 33–34 Weeks	**1.226**	**1.063**	**1.414**	**1.178**	**1.004**	**1.383**
Birth weight	<2500 grams vs. ≥2500 grams	0.879	0.636	1.216	0.918	0.634	1.329
	missing vs. ≥2500 grams	1.043	0.74	1.47	0.977	0.659	1.449
Birth type	Multiplets vs. Singleton	0.944	0.815	1.094	**0.813**	**0.686**	**0.963**
	Unknown vs. Singleton	0.86	0.7	1.057	**0.763**	**0.602**	**0.966**
NICU during birth admission	Characteristic Present vs. Absent	**1.384**	**1.122**	**1.708**	**1.294**	**1.012**	**1.653**
CLDP	Characteristic Present vs. Absent	**2.033**	**1.789**	**2.311**	**1.89**	**1.635**	**2.186**
CHD	Characteristic Present vs. Absent	**1.494**	**1.275**	**1.751**	**1.551**	**1.292**	**1.86**
Comorbidity other than CHD, CLDP	Characteristic Present vs. Absent	**1.407**	**1.252**	**1.581**	**1.511**	**1.323**	**1.727**
RSV-related admission prior to season	Characteristic Present vs. Absent	**1.439**	**1.128**	**1.835**	3.531	0.806	15.47
Inpatient admission or emergency room visit prior to first dose	Characteristic Present vs. Absent	**2.521**	**2.253**	**2.82**	**1.727**	**1.531**	**1.948**

Bold indicates statistically significant results.

*Excluding infants with any RSV-related admission that occurred prior to prior to first palivizumab dose AND prior to November 30.

^†^Infants with gestational age >34 weeks also had either CLDP or CHD.

*CHD*: Congenital heart disease; *CLDP*: Chronic lung disease of prematurity; *NICU*: Neonatal intensive care unit; *ED*: Emergency department.

## Discussion

This is the largest study to date examining the association between partial prophylaxis and RSV-related hospitalizations among Medicaid infants who received palivizumab. Two-thirds (66.5%) of the high-risk infants in our study received partial prophylaxis with palivizumab. Approximately one in every five infants failed to initiate palivizumab dosing until after November 30^th^.

The percentage of infants with partial prophylaxis in our study is consistent with noncompliance rates previously reported for the Medicaid population [2, 3, 10]. Hampp et al. analyzed palivizumab utilization and compliance in children less than 2 years of age covered under the fee-for-service Florida Medicaid program. During the 2004–2005 RSV season, 67.9% of palivizumab recipients were compliant, defined by the presence of at least 4 claims for the drug from October through February [10]. Compliance decreased to 41.3% with the requirement for a minimum of 5 doses. Furthermore, approximately 33% of ≤32 wGA infants in that study received no in-season palivizumab doses, which suggests that many high risk infants are unprotected while virus circulation is highest. Diehl et al. documented a 29.8% compliance rate during the 2006–2007 RSV season based on number and timing of doses in a population of infants (59.2% Medicaid) drawn from a Pennsylvania managed care plan [3]. A review by Frogel et al. of palivizumab compliance documented variability in measurement and rates across published studies [2]. They found that compliance with palivizumab dosing was higher in home health programs compared to office settings, which translated to improvements in health outcomes among infants in the former group.

Compliance with prophylaxis was previously shown to be higher in children from nonsmoking families, those whose parents believed palivizumab would have a positive effect, and those whose parents did not report difficulty with transportation [2]. The design of our study did not allow for the evaluation of those specific factors but we did find a strong association between partial prophylaxis and capitated plan membership. According to Centers for Medicare & Medicaid services, in 2010, 54,612,393 individuals were enrolled in managed Medicaid plans. This is 71.5% of total enrollment, and a 25.8% increase over 2001 (56.8%) [14]. This trend toward managed care underscores the importance of understanding why palivizumab dosing in high-risk Medicaid infants is a particular challenge in capitated health plans.

Our study also found potential disparities in palivizumab use between racial/ethnic minorities and Caucasians, including number and timing of doses, and within each ethnic group, infants in capitated plans were more likely to be partially prophylaxed. Low-socioeconomic status, limited parental knowledge of RSV and the efficacy of RSV prophylaxis, and the quality of communication between healthcare professionals and parents of high-risk infants may potentially contribute to the observed palivizumab utilization patterns and also may potentially influence use of inpatient care.

The current study provides further insight into the risk of RSV hospitalization in high-risk infants in Medicaid. Although previously published data generally show that compliance is associated with decreased hospitalization rates, study designs and the estimated association vary [2]. Analysis of data from the Palivizumab Outcomes Registry by Frogel et al. showed a significantly lower risk for RSV hospitalization (OR 0.702, 95% CI 0.543-0.913) in patients who were compliant, defined by number of doses and dosing intervals, but found no association using a compliance definition based only on number of doses [6]. In that study, a higher risk for RSV hospitalization was also found for Medicaid versus non-Medicaid patients. By contrast, Diehl et al. found no significant differences between compliant and noncompliant infants in RSV hospitalization, but this finding may have been impacted by the small sample size (N=245) [3]. Using time-dependent exposure definitions to accommodate intermittent palivizumab dosing, Winterstein et al. in a study of Florida Medicaid children found decreases in the risk of RSV hospitalization subsequent to both the initial palivizumab dose and succeeding doses [15]. However, the reduction following the first dose [HR 0.89, 95% CI 0.71-1.12] was not statistically significant. The risk reduction associated with subsequent doses (HR 0.56, 95% CI 0.46-0.69), however, was similar to the lower range of results reported in palivizumab trials [8, 9].

We found a higher rate of RSV-related hospitalization (7.9% among fully prophylaxed infants) compared to the 4.8% rate in the IMpact-RSV trial [8]. There are a number of possible explanations for this difference, including increased awareness of the risks of RSV and increased monitoring in the trial population. The background RSV incidence is likely to be greater in the Medicaid population than in the clinical trial populations. Sangare et al. reported that infants covered by California Medicaid were twice as likely to be hospitalized with RSV versus infants covered under other insurance [relative risk (RR) 2.03, 95% CI 1.99-2.06] [7]. In addition, the high prevalence of comorbidities (56% of infants overall) in our study population and the use of diagnosis codes beyond simply RSV may have also contributed to the higher hospitalization rate. Our decision to use the expanded code list was driven by an acknowledgement that RSV-specific ICD-9-CM codes are underutilized in practice. Our RSV-related rates are within range of those reported by Boyce et al. who calculated RSV hospitalization rates based on a definition inclusive of RSV infection and bronchiolitis and found rates of 57 – 388 per 1,000 Tennessee Medicaid children less than 1 year of age [5].

We observed that a substantial proportion of RSV-related hospitalizations occurred prior to the first palivizumab dose. This finding suggests missed opportunities for prevention. In a subgroup analysis, omitting these infants with RSV-related hospitalizations prior to first dose did not alter the finding of increased risk of hospitalization among infants with partial prophylaxis.

Our study also found differences in the severity of RSV-related hospitalization for fully and partially prophylaxed infants. Partially prophylaxed infants had longer RSV-related hospital stays and a higher proportion of these infants were admitted to an ICU or received mechanical ventilation or supplemental oxygen compared with fully prophylaxed infants. Our findings are aligned with the secondary clinical efficacy endpoints from the IMpact RSV Clinical Study, which also found significant differences in length of RSV hospitalization stay and ICU admissions among the palivizumab group compared with placebo group [8]. In addition, a recent study found an average of 1.4 fewer days in the hospital among RSV-prophylaxed infants compared to infants without RSV prophylaxis [16]. Future studies should focus on the economic benefits associated with reducing both the incidence and severity of RSV disease in the hospital setting with complete palivizumab dosing.

There are several limitations to these analyses. Administrative claims are collected for payment purposes and not clinical research and therefore are subject to coding errors, which may impact identification of clinical outcomes. In addition, claims do not capture data on socioeconomic factors, distance from medical facilities and other factors that may shape utilization patterns. Owing to the non-randomized nature of the study, demographic differences between groups such as prior hospitalization use or proportion with CLDP and CHD could impact the results. However, after multivariate adjustment and subgroup specific analyses, the treatment effect remained, suggesting that these differences may not have a major effect. Palivizumab doses administered to an infant during a hospitalization are not captured separately on the hospital claim. Therefore, it was necessary to exclude subjects born during the RSV season since there was a high likelihood that not all palivizumab doses received by these infants would appear in the data. Although this approach ensures greater accuracy for our palivizumab compliance measures, it is possible the RSV-related hospitalization risk may be underestimated. Our study may over- or underestimate severe RSV disease because we did not have RSV test results and had to rely on the diagnosis codes for RSV as well as unspecified bronchiolitis and pneumonia. We believe this is a reasonable approach given known low rates of RSV testing which stems in part from the American Academy of Pediatrics recommendations that routine testing is not required once the RSV season has started because it rarely alters clinical management [17]. Given that the MarketScan® Medicaid Multi-State Database includes enrollees from geographically dispersed states, we were not able to accurately define the start and length of the RSV season. By not incorporating the variance of seasons in different regions in the U.S., there could be misclassification of cases using our algorithm. Finally, this study analyzed Medicaid patients only and results are not generalizable to a commercially insured population.

## Conclusions

This is the largest study to date to examine the association of partial palivizumab prophylaxis with RSV-related hospitalization in a Medicaid population. Our findings support starting palivizumab dosing prior to the start of the RSV season and ensuring complete and timely dosing throughout the season in order to optimize protection against RSV. Our findings also show that RSV prophylaxis dosing regimens other than FDA-labeled recommendations of monthly throughout the RSV season may be associated with greater risk of RSV-related hospitalization. Furthermore, a substantial number of infants experienced an RSV-related hospitalization prior to administration of the first dose, suggesting a missed opportunity to help prevent disease. Future research should examine barriers to care for palivizumab use, the impact of healthcare disparities on palivzumab prophylaxis and RSV hospitalization, and the potential clinical and economic benefits associated with prophylaxis.

## Electronic supplementary material

Additional file 1:**Codes Used to Identify Comorbid Conditions.**(PDF 156 KB)

Additional file 2:**Distribution of Comorbid Conditions in Study Population.**(PDF 108 KB)

## Abbreviations

RSV: Respiratory syncytial virus
CLDP: Chronic lung disease of prematurity
CHD: Congenital heart disease
NICU: Neonatal intensive care unit.
