# Supplementary material for: Partial palivizumab prophylaxis and increased risk of hospitalization due to respiratory syncytial virus in a Medicaid population: a retrospective cohort analysis
Source: BMC Pediatr. 2014 Oct 13;14:261. doi: 10.1186/1471-2431-14-261 (PMC4287588; doi:10.1186/1471-2431-14-261)
Supplement: Supplementary file 1 — Additional file 1:Codes Used to Identify Comorbid Conditions.(PDF 156 KB) [file 12887_2013_1220_MOESM1_ESM.pdf]

Additional File 1: Codes Used to Identify Comorbid Conditions

Comorbid Conditions	
**Description**	**ICD-9-CM Diagnosis Code**
Congenital Heart Disease	424.x,.425.3x, 425.4x, 425.8x, 745.xx – 747.xx
Chronic Lung Disease	770.7x,227.02,333.71,356.1,358.1,747.83,748.4, 748.5,748.6,750.3,756.6,770.12,770.81,770.82, 240-242.xx,243,244.x,246.1,277.81,277.82, 335.0,335.10,335.11,335.19,335.21,343.x,359.0, 359.1,359.21,359.22,359.6-359.7x,446.5 551.3,552.3,553.3,710.3,756.6,769,770.0,770.7x, 775.3
Muscular dystrophy	359.0x-359.1x.
Anterior horn cell disease	335.xx
Intraventricular hemorrhage	772.1x
Immunodeficiency	279.x
HIV	042.xx, V08.xx, 079.51-079.53
Sickle Cell Anemia	282.4x-282.6x
Hydrocephalus	742.3x, 741.0x
Periventricular leukomalacia	779.7x
Necrotizing enterocolitis	777.5x
Trisomy 21	758.0
Cerebral palsy	343.xx, 333.71
Other neuromuscular, immunological and genetic conditions	740.0x-759.x (except for codes in this range that are classified elsewhere), 246.1,258.1, 356.1, 277.81, 277.82,446.5,551.3,335.0, 335.10,335.11,335.19,710.3
Cystic Fibrosis	277.0x
Retinopathy of prematurity	362.21, 362.29
Failure to Thrive	783.4x
**Description**	**ICD-9-CM Procedure Code**
Congenital Heart Disease	35.41
Chronic Lung Disease	V46.2
**Description**	**CPT Codes**
Congenital Heart Disease	92992, 92993
Chronic Lung Disease	4030F
**Description**	**Revenue Codes**
Chronic Lung Disease	0277, 060x
**Description**	**HCPCS Codes**
Chronic Lung Disease	E0424-E0444, E0455, E1353, E1390, E1391, K0738
**Description**	**Medication Name (Generic)**

Congenital Heart Disease	Acebutolol Hydrochloride, Amiloride Hydrochloride, Amiloride Hydrochloride/Hydrochlorothiazide, Amlodipine Besylate/Benazepril Hydrochloride, Atenolol, Atenolol/Chlorthalidone, Benazepril Hydrochloride, Benazepril Hydrochloride/Hydrochlorothiazide, Bendroflumethiazide, Bendroflumethiazide/Nadolol, Bendroflumethiazide/Rauwolfia Serpentina, Benzthiazide, Betaxolol Hydrochloride, Bisoprolol Fumarate, Bisoprolol Fumarate/Hydrochlorothiazide, Bumetanide, Candesartan Cilexetil, Candesartan Cilexetil/Hydrochlorothiazide, Captopril, Captopril/Hydrochlorothiazide, Carteolol Hydrochloride, Carvedilol, Chlorothiazide, Chlorothiazide Sodium, Chlorothiazide/Methyldopa, Chlorothiazide/Reserpine, Chlorthalidone, Chlorthalidone/Clonidine Hydrochloride, Chlorthalidone/Reserpine, Cyclandelate, Deserpidine/Hydrochlorothiazide, Deserpidine/Methyclothiazide, Diazoxide, Digoxin, Digoxin Immune Fab (ovine), Diltiazem Malate/Enalapril Maleate, Enalapril Maleate, Enalapril Maleate/Felodipine, Enalapril Maleate/Hydrochlorothiazide, Enalaprilat, Epoprostenol Sodium, Eprosartan Mesylate, Eprosartan Mesylate/Hydrochlorothiazide, Esmolol Hydrochloride, Ethacrynic Acid, Ethaverine Hydrochloride, Ethaverine/Apap/Salicyl/Atrop, Ethaverine/B-complex, Fenoldopam Mesylate, Fosinopril Sodium, Fosinopril Sodium/Hydrochlorothiazide, Furosemide, Guanethidine Monosulfate/Hydrochlorothiazide, HCTZ/Hydralazine HCl/Reserpine, Hydralazine Hydrochloride, Hydralazine Hydrochloride/Hydrochlorothiazide, Hydralazine Hydrochloride/Isosorbide Dinitrate, Hydralazine Hydrochloride/Reserpine, Hydrochlorothiazide, Hydrochlorothiazide/Irbesartan, Hydrochlorothiazide/Labetalol Hydrochloride, Hydrochlorothiazide/Losartan Potassium, Hydrochlorothiazide/Methyldopa, Hydrochlorothiazide/Metoprolol Tartrate, Hydrochlorothiazide/Moexipril Hydrochloride, Hydrochlorothiazide/Olmesartan Medoxomil, Hydrochlorothiazide/Propranolol Hydrochloride, Hydrochlorothiazide/Quinapril Hydrochloride,

	Hydrochlorothiazide/Reserpine, Hydrochlorothiazide/Spironolactone, Hydrochlorothiazide/Telmisartan, Hydrochlorothiazide/Timolol Maleate, Hydrochlorothiazide/Triamterene, Hydrochlorothiazide/Valsartan, Hydroflumethiazide, Hydroflumethiazide/Reserpine, Indapamide, Irbesartan, Kcl/Bendroflumethiazide, Labetalol Hydrochloride, Lisinopril, Lisinopril/Hydrocholorothiazide, Losartan Potassium, Methyclothiazide, Methyclothiazide/Reserpine, Metolazone, Metoprolol Succinate, Metoprolol Tartrate, Minoxidil, Moexipril Hydrochloride, Nadolol, Nylidrin Hydrochloride, Olmesartan Medoxomil, Papaverine Hydrochloride, Papaverine/Codeine, Penbutolol Sulfate, Perindopril Erbumine, Pindolol, Polythiazide, Polythiazide/Prazosin Hydrochloride, Polythiazide/Reserpine, Propranolol Hydrochloride, Quinapril Hydrochloride, Ramipril, Reserpine/Trichlormethiazide, Spironolactone, Telmisartan, Timolol Maleate, Tolazoline Hydrochloride, Torsemide, Trandolapril, Trandolapril/Verapamil Hydrochloride, Treprostinil Sodium, Triamterene, Trichlormethiazide, Trimethaphan Camsylate, Valsartan
Chronic Lung Disease	arformoterol, formoterol, salmeterol, fluticasone/salmeterol, budesonide/formoterol, albuterol, bitolterol, epinephrine, isoetharine, isoproterenol, levalbuterol, metaproterenol, pirbuterol, terbutaline, aminophylline, dyphylline, oxtriphylline, theophylline, ipratropium, bendroflumethiazide, benzthiazide, chlorothiazide, chlorthalidone, hydrochlorothiazide (HCTZ), hydroflumethiazide, indapamide, methyclothiazide, metolazone, polythiazide, trichlormethiazide, bumetanide, ethacrynic acide, furosemide, torsemide, amiloride, spironolactone, triamterene, atenolol/chlorthalidone, bisoprolol/HCTZ, metoprolol/HCTZ, nadolol/bendroflumethiazide, propranolol/HCTZ, timolol/HCTZ, benazepril/HCTZ, captopril/HCTZ, enalapril/HCTZ, fosinopril/HCTZ, lisinopril/HCTZ, moexipril/HCTZ, quinapril/HCTZ, candesartan/HCTZ, eprosartan/HCTZ, irbesartan/HCTZ, losartan/HCTZ, olmesartan/HCTZ, telmisartan/HCTZ, valsartan/HCTZ, amiloride/HCTZ,

	spironolactone/HCTZ, triamterene/HCTZ, prazosin/polythiazide, clonidine/chlorthalidone, dexamethasone, methylprednisolone,
