# Supplementary material for: Partial palivizumab prophylaxis and increased risk of hospitalization due to respiratory syncytial virus in a Medicaid population: a retrospective cohort analysis
Source: BMC Pediatr. 2014 Oct 13;14:261. doi: 10.1186/1471-2431-14-261 (PMC4287588; doi:10.1186/1471-2431-14-261)
Supplement: Supplementary file 2 — Additional file 2:Distribution of Comorbid Conditions in Study Population.(PDF 108 KB) [file 12887_2013_1220_MOESM2_ESM.pdf]

Additional File 2: Distribution of Comorbid Conditions in Study Population

**Comorbid Conditions**	Compliant Infants (N=2,828)		Noncompliant Infants (N=5,615)	
Any Congenital Disease (N, %)	1,572	55.6%	3,164	56.3%
Muscular dystrophy	1	0.0%	7	0.1%
Anterior horn cell disease	0	0.0%	0	0.0%
Intraventricular hemorrhage	397	14.0%	783	13.9%
Immunodeficiency	8	0.3%	37	0.7%
HIV	0	0.0%	2	0.0%
Sickle cell anemia	16	0.6%	22	0.4%
Hydrocephalus	45	1.6%	123	2.2%
Periventricular leukomalacia	31	1.1%	65	1.2%
Necrotizing enterocolitis	131	4.6%	356	6.3%
Trisomy 21	36	1.3%	116	2.1%
Cerebral palsy	14	0.5%	22	0.4%
Other neuromuscular, immunological and genetic conditions	237	8.4%	590	10.5%
Cystic Fibrosis	6	0.2%	24	0.4%
Retinopathy of prematurity	1,085	38.4%	2,115	37.7%
Failure to thrive	269	9.5%	622	11.1%
CHD	195	6.9%	606	10.8%
CLDP	384	13.6%	858	15.3%
